# Intraoperative fluorescence angiography with indocyanine green: retrospective evaluation and detailed analysis of our single-center 5-year experience focused on colorectal surgery

**DOI:** 10.1515/iss-2020-0009

**Published:** 2020-09-18

**Authors:** Christoph Marquardt, Georgi Kalev, Thomas Schiedeck

**Affiliations:** Department of General, Visceral, Thoracic and Pediatric Surgery, Ludwigsburg Hospital, Ludwigsburg, Baden-Wuerttemberg, Germany

**Keywords:** anastomotic leakage, colorectal surgery, fluorescence angiography, fluorescence imaging, indocyanine green, perfusion

## Abstract

**Objectives:**

Assessing bowel perfusion with indocyanine green fluorescence angiography (ICG-FA) shows positive effects on anastomotic healing in colorectal surgery.

**Methods:**

A retrospective evaluation of 296 colorectal resections where we performed ICG-FA was undertaken from January 2014 until December 2018. Perfusion of the bowel ends measured with ICG-FA was compared to the visual assessment before and after performing the anastomosis. According to the observations, the operative strategy was confirmed or changed. Sixty-seven low anterior rectal resections (LARs) and 76 right hemicolectomies were evaluated statistically, as ICG-FA was logistically not available for every patient in our service and thus a control group for comparison resulted.

**Results:**

The operative strategy based on the ICG-FA results was changed in 48 patients (16.2%), from which only one developed an anastomotic leakage (AL) (2.1%). The overall AL rate was calculated as 5.4%. Within the 67 patients with LAR, the strategy was changed in 11 patients (16.4%). No leakage was seen in those. In total three AL happened (4.5%), which was three times lower than the AL rate of 13.6% in the control group but statistically not significant. From the 76 right hemicolectomies a strategy change was undertaken in 10 patients (13.2%), from which only one developed an AL. This was the only AL reported in the whole group (1.3%), which was six times lower than the leakage rate of the control group (8.1%). This difference was statistically significant (p=0.032).

**Conclusions:**

Based on the positive impact by ICG-FA on the AL rate, we established the ICG-FA into our clinical routine. Although randomized studies are still missing, ICG-FA can raise patient safety, with only about 10 min longer operating time and almost no additional risk for the patients.

## Introduction

During surgical procedures, the use of any technology providing patient safety is self-evident. Since 2014, we used indocyanine green fluorescence angiography (ICG-FA) for visualizing the perfusion of bowel ends before and after performing the anastomosis, especially in colorectal surgery. Due to our positive first clinical results with less anastomotic leakage (AL) rates in selected patient groups (rectal resections, [1]), we started to use ICG-FA on any patient who underwent a bowel resection. As ICG-FA was not available and thus not used on every possible patient during the investigated period of time, we collected those patients who did not undego ICG-FA for comparison as our own control group. For the following two groups, a control group from our own patients was available for statistical evaluation: right hemicolectomy and anterior rectal resection. In this work, we retrospectively evaluated our patient data of the last 5 years (2014–2019).

## Materials and methods

From January 2014 until December 2018, we used ICG-FA during the operations of 340 patients. From these, we especially evaluated different bowel resections (elective and emergency procedures), including 296 colorectal resections (open, laparoscopic or robotic anterior resection of the rectum, Hartmann reversal, oncological transverse colon resection, left hemicolectomy, right hemicolectomy, tubular resection for diverticulitis and resection rectopexy). Further, we used ICG-FA to assess the bowel perfusion in patients with ischemic mesenterial disease of embolic, thrombotic or septic origin, or in patients with strangulated intestine through incarcerated hernia. Patients with malignant disease were treated in the context of the national guidelines and according to international standard protocols [2], [3].

**Figure 1:**
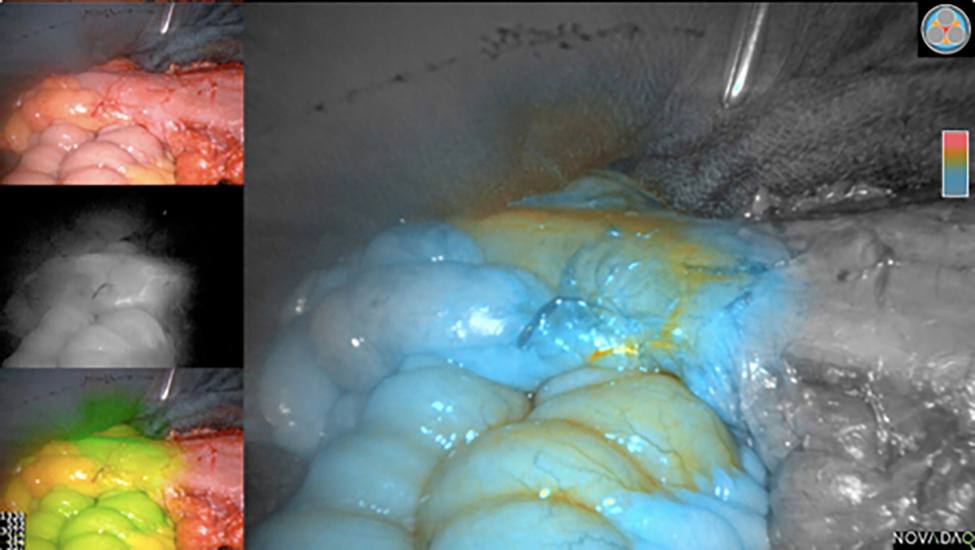
ICG-FA before dissection of the descending colon. Instrument pointing at the site of dissection in the well perfused area (orange).

One day before colorectal surgery bowel preparation (one package of Moviprep^®^ (Norgine GmbH, Wettenberg, Germany) with 1 L of fluid orally) and selective oral bowel decontamination (standard solution with Colistin and Tobramycin, manufactured at our in hospital pharmacy) was the standard of care. Further 30–60 min before the operation, an intravenous single-shot antibiotic prophylaxis with a cephalosporine of the 2nd or 3rd generation plus metronidazole was administered. Intraoperatively, the perfusion was first assessed by the surgeon due to widely discussed visual criteria, and the proposed dissection line was marked directly on the bowel [4]. Then perfusion was visualized by ICG-FA in the following way: after complete dissection and just before resection an ICG standard dose of 0.1–0.2 mg/kg body weight of Verdeye® (Diagnostic Green GmbH, Aschheim-Dornach, Germany) was given intravenously. We used the SPY System (Novadaq Technologies, Concord, ON, Canada) with laparoscopic optics (0 and 30 degree angle) for open and laparoscopic surgery and Firefly (Intuitive Surgical, Sunnyvale, Kalifornien, USA), the last mentioned only during robotic surgery. These systems provide qualitative real-time fluorescence imaging of the bowel perfusion in the visible and near-infrared spectrum. As quantitative parameters are not provided, the perfusion was assessed subjectively based on our experience. We follow the protocol of Thöns [5], who used a loop of the small intestine as a reference for perfusion by holding it into the observed region of interest for comparison. Thus the increasing intensity of the flooding ICG in the bowel, which has to be resected, compared with the small bowel loop and finally the resulting peak intensity after some seconds could be observed and video recorded. We compared the result to our clinical evaluation and noted any strategic change, especially change of the before marked dissection line. The bowels were dissected in the well perfused region, that showed saturated green in the ICG-FA (Figure 1) and then the anastomosis was performed. Then standard tests were used to control the result, circumferential visual control, air leak test (ALT) and donut assessment for circular integrity when a circular stapler had been used. Then the perfusion of the anastomosis was assessed first visually by the surgeon and then with ICG-FA. Every change of our operative strategy (e.g. change of resection line, additional sutures, resection and remanufacturing of the anastomosis, diverting ileostomy even though not planned preoperatively or abandonment of diverting ileostomy even though planned) was noted.

As we retrospectively investigate the influence of a technical method for visualization of perfusion of bowel resection lines before and after performing an anastomosis on our key end point “anastomotic healing rate”, we had to stratify our patients in groups due to surgical criteria influencing bowel perfusion (anatomical resection type, central ligation of vessels, tubular resection) and patient criteria influencing anastomotic healing (sex, BMI, American Society of Anesthesiologists [ASA], malignant disease, staging according to the Union for International Cancer Control [UICC], infection/diverticulitis, diverting ileostomy, elective/emergency surgery, open/laparoscopic/robotic, technique of anastomosis, ALT, etc). For the following procedures and patient groups, our own data from the same time period delivered a control group for statistical evaluation.

### Right hemicolectomy

As we regularly follow the principles of complete mesocolic excision (CME) ([6], open book technique), dissecting along the embryonic planes is crucial to completely remove the whole mesocolon with all possible lymphatic metastases. We dissect along the superior mesenteric vein and superior mesenteric artery, centrally ligate the ileocolic artery and vein and the right colonic vein and artery (if present). In case of extended right hemicolectomy, besides the before mentioned, the middle colonic vein and artery (A. and V. colica media) are centrally ligated too.

### Rectal resections

In rectal resections for patients with cancer, we differentiated between high anterior rectal resection (HAR) with proximal mesorectal excision (PME) and low anterior rectal resection (LAR) with total mesolectal excision (TME), which are evaluated in common and separately. Patients went through neoadjuvant radiochemotherapy (nRCTx) in low rectal cancer for stages T3/T4 or with suspicious lymphnodes (N+), diagnosed by endosonographic ultrasound or magnetic resonance imaging.

**Figure 2:**
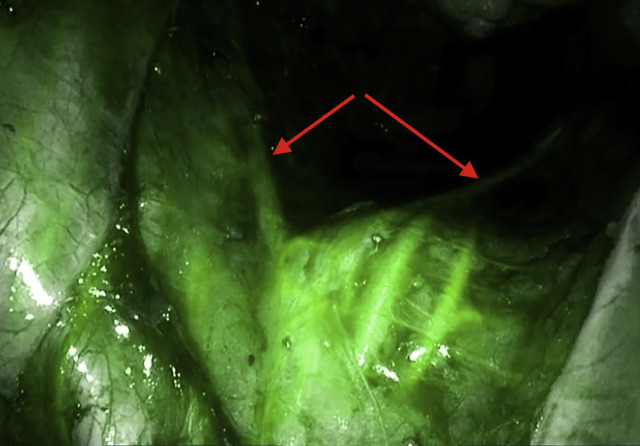
Hypogastric plexus, left and right branches of the hypogastric nerve at the promontorium (red arrows).

Technically we follow the national guidelines by routinely performing a mobilization of the splenic flexure and a “high-tie” of the inferior mesenteric artery and inferior mesenteric vein [3]. Further PME and TME are performed in a nerve sparing manner [2] respecting the “holy plane” [7] and visualizing the hypogastric plexus by ICG-FA (Figure 2). The TME was executed down to the pelvic floor for resection of the whole mesorectum. Then the rectum was transected below the mesorectum with a linear stapler. The deep anastomosis was performed side-to-end in double stapling technique with a circular stapler of a minimum diameter of 28 mm. The stapler anvil was fixed with a purse string suture. Integrity of the donuts and ALT for AL was always performed and a diverting ileostomy marked on all patients preoperatively [1].

As the surgical approach does not influence the bowel perfusion, nor the anastomotic healing rate, conventionally open, laparoscopic and robotic resections were evaluated together. To compare our data with the literature, we assessed any AL according to the International Study Group of Rectal Cancer (Grade A–C) [8].

## Results

From January 2014 until December 2018, we used ICG-FA on different patients during elective and emergency procedures. During colorectal resections of 296 patients, ICG-FA procedure was performed. On 48 of those the intraoperative strategy was changed (16.2%) due to the results of ICG-FA. Thus on 26 patients the resection line was changed, on 16 patients a planned diverting stoma was omitted, one patient received a diverting stoma even not planned preoperatively, on one patient the resection line was changed and a non planned diverting stoma performed and on four patients the anastomosis was secured by additional sutures (Table 1). In this group only one patient suffered from an AL (2.1%) after right hemicolectomy.

**Table 1:** Change of intraoperative strategy due to ICG-FA during colorectal resections while performing the anastomosis and resulting AL rate.

Type of strategy change	Number of patients	AL
Change of resection line	26	1
No stoma even though planned	16	0
Stoma even though not planned	1	0
Change of resection line plus ostomy even though not planned	1	0
Additional sutures of anastomosis	4	0
Total	48	1 (2.1%)

ICG-FA, indocyanine green fluorescence angiography.

Those 296 patients included the following resection types: 67 (22.6%) LAR plus TME for rectal cancer and 76 (25.7%) right hemicolectomies with CME and ileotransversostomy for cancer, for which we had a control group. 57 (19.3%) HAR plus PME for sigmoid cancer, 24 (8.1%) oncological transverse colon resection with a handsewn end-to-end anastomosis for transverse or flexure carcinoma, 7 (2.4%) Hartmann reversal procedures, 53 (17.9%) tubular left hemicolectomies for diverticular disease and 12 (4.1%) resection rectopexies. For the at last mentioned resections we had no control group for statistical analysis and thus they were not further evaluated.

In the following we evaluated two homogenous groups of patients, where our own data of patients operated without using ICG-FA were available for comparison: right colon cancer and cancer of the middle and lower third of the rectum (according to UICC, where the lower rectum is measured 0–6 cm orally from the anal verge and the middle third 6 to <12 cm from the anal verge). Here the above mentioned technical operative steps were equally performed on every cancer patient, permitting a reliable comparison and statistical evaluation.

Sixty-seven patients (32 female, 35 male) with a rectal cancer in the middle or lower third of the rectum were operated by standardized LAR with TME using ICG-FA. Fifty-nine equally treated patients (22 female, 37 male) were used as control group, as they were operated without using ICG-FA. All data of both groups (age, BMI, ASA, UICC) are shown in Table 2.

**Table 2:** Clinical parameters of patients after low anterior rectal resection.

	Sex f/m	Age	ASA				Open /lap	Diverting ileostomy	UICC					BMI	RTx
			I	II	III	IV			0	I	II	III	IV		
Control group	22/37	71(60–78)^b^	2	28	28	1	36/23	49 (83.1%)	3	19	14	14	9	26.5^a^ (23.9–31.1)^a^	32 (54.2%)
ICG-FA group	32/35	69^a^ (58–76)^b^	0	44	22	1	26/41	56 (83.6%)	6	15	18	20	8	25.2^a^ (23–27.6)^b^	31 (46.3%)

^a^Median.

^b^First and third quartile.

ICG-FA, indocyanine green fluorescence angiography; ASA, American Society of Anesthesiologists; UICC, staging according to the Union for International Cancer Control.

Between the groups there was no statistically significant difference regarding BMI, age, ASA, UICC, sex, nRCTx and diverting ileostomy (Table 3).

**Table 3:** Statistical verification of differences between ICG-FA and control group after rectal resections.

	BMI	Age	ASA	UICC	Sex	RTx	Diverting ileostomy
Significance	p=0.07	p=0.37	p=0.09	p=0.75	p=0.24	p=0.37	p=0.94

ICG-FA, indocyanine green fluorescence angiography; ASA, American Society of Anesthesiologists; UICC, staging according to the Union for International Cancer Control.

In the ICG-FA group the intraoperative strategy was changed on 11 of the 67 patients (16.4%) due to ICG-FA, none of those showed an AL. One male with low anastomosis (4 cm orally from the anal verge) did not receive a diverting ileostomy, whereupon an AL of grade C occurred. Two other patients showed an AL: one of grade A, one of grade C, resulting in an overall leakage rate of 4.5%. In the control group (Table 2) eight AL occurred (13.6%), one of grade A, one of grade B and six of grade C.

Despite observing a three times lower AL rate in the ICG-FA than the control group (4.5% vs. 13.6%), the statistical tests delivered no significance (p=0.068).

Standard right hemicolectomy with CME and ICG-FA was performed on 76 patients (45 female, 31 male) with a right colon cancer. Patient data including age, ASA, BMI and UICC stage are listed in Table 4. Only one AL happened and the AL rate was calculated as 1.3%. The intraoperative strategy was changed in 10 of those 76 patients (13.2%) due to ICG-FA and in this group only one single AL of grade C occurred. In this patient, ICG-FA showed a low perfusion of the distal ileum, and the resection line was changed. Despite that change, a point-like AL happened and a revision laparotomy had to be performed.

**Table 4:** Clinical parameters of patients after right hemicolectomy.

	Sex f/m	Age	ASA				BMI	UICC			
			I	II	III	IV		I	II	III	IV
Control group	74/75	77^a^ (69–82)^a^	2	46	79	22	26^a^ (23–29.6)^b^	25	63	39	22
ICG-FA group	45/31	74^a^ (65–80)^b^	1	42	28	5	25.8^a^ (21.7–29.3)^b^	10	37	14	9

^a^ Median.

^b^ First and third quartile.

ICG-FA, indocyanine green fluorescence angiography; ASA, American Society of Anesthesiologists; UICC, staging according to the Union for International Cancer Control.

During the same period of time, 149 patients (74 female, 75 male) with right colon cancer were treated equally by right hemicolectomy with CME without using ICG-FA (Table 4).

Between the two groups, no significant difference regarding age, sex, BMI and UICC stage was seen (Table 5). The ASA score showed significantly more ASA III and IV patients in the control group.

**Table 5:** Statistical verification of differences between ICG-FA and control group after right hemicolectomy.

	BMI	Age	ASA	UICC	Sex
Significance	p=0.68	p=0.06	p=0,001	p=0.25	p=0.18

ICG-FA, indocyanine green fluorescence angiography; ASA, American Society of Anesthesiologists; UICC, staging according to the Union for International Cancer Control.

In the control group 12 AL of grade C were reported, leading to an AL rate of 8.1%, which was six times higher than the ICG-FA group of 76 patients with only one AL (1.3%). The statistical evaluation using Fischer’s exact test a significant result was confirmed (p=0.032).

Summing up all 296 colorectal resections where we used ICG-FA, we observed 16 AL leading to an overall AL rate of 5.4% for our patients.

## Discussion

The AL rate in the literature differs between 1 and 21% [9], [10], [11]. This broad range of AL rates results from the different definitions of AL, different risk factors, entities, indications for operations and different localizations of the anastomosis. Underlying malignant diseases lead to a higher AL rate [9]. For the ileocolostomy as part of a right hemicolectomy, the AL rate is reported between 1% and 8.4% [9], [12]. For LAR, the AL rate varies between 3 and 21% [11], [13], [14]. Latest German nationwide evaluation of 5,77,325 colorectal resections give an overall AL rate of 6.6% [15], where our own AL rate from colorectal resections with the use of ICG-FA lies with 5.4% below that level. Further in all our reported patient groups (with and without ICG-FA), the observed AL rates of our own patients lie in those reported limits.

The etiology of AL depends on many factors. Male sex, ASA≥III, cigarette smoking, alcohol consumption, intraoperative blood transfusion, no protective diverting stoma, higher UICC stages, and low rectal cancer were proven to be independent risk factors for AL in a multicenter German prospective study [16]. Further risk factors are high age, deep rectal anastomosis, underlying malignant disease, higher ASA score, long operation time, emergency procedure and nRCTx [17]. Although nRCTx is generally regarded as risk factor for AL and many retrospective studies show a direct correlation between nRCTx and AL [11], [13], Sebag-Montefiore et.al. [18] reported no difference in AL rate between nRCTx and selective postoperative CTx. A meta-analysis from 2017 showed no negative influence of nRCTx on the AL rate after LAR for rectal cancer of the middle and lower third [19]. The same resulted from a propensity score matching analysis from 2014 [20].

A protective diverting ileostomy was regularly planned in any LAR, as it was shown to prevent AL after LAR [21], [22] and severe septic complications and operative revisions due to AL were reduced [23]. Due to our national guidelines and in our understanding, the above mentioned AL rates represent a high risk for the patient, which has to be lowered by any means, e.g., performing a protective ileostomy.

Our patient groups contained all different risk factors. The groups were homogenous and comparable for statistical evaluation (Tables 3, 5), as only for ASA in right hemicolectomies a statistically higher number of ASA III and IV was noted in the control group (Table 5).

Adequate perfusion of the anastomosed bowel ends is regarded crucial for optimal anastomotic healing. Low tissue oxygenation based on inadequate perfusion was shown to play an important role for the development of an AL [24]. The commonly used technique for the assessment of local bowel perfusion is the subjective clinical estimation by the operating surgeon. The subjective criteria: color of serosa and mucosa, bleeding of the bowel ends, pulsation of nearby small vessels and temperature are strongly influenced by the individual experience of the surgeon and other external factors. One additional tool for semiquantitative assessment of the bowel perfusion is delivered by the use of ICG-FA. More and more ICG-FA is accepted as a proper mean for assessment of bowel perfusion and thus reduction of the AL rate. The subject of numerous studies is the change in operative strategy after the intraoperative use of ICG-FA. In our 296 colorectal resections, the strategy was changed in 48 patients (16.2%) and only one AL happened after right hemicolectomy. For all other procedures, where ICG-FA changed the strategy in 8.3% of our 24 transverse colon resections with handsewn colocolic anastomosis, in 13.2% of our 53 tubular sigmoid resections for diverticulitis, in 28.6% of our Hartmann reversal procedures, in 16.7% of our 12 resection rectopexies and in 21% of our 57 HAR with PME, no leakage occurred. We regard this fact as a very hard criterion in favor of the use of ICG-FA. In the literature the strategy change was reported between 2.5 and 20% [25]. Morales–Conde concluded a maximum impact for ICG-FA on the left-sided resections, where he noted significantly more strategy changes [26]. Summing up our left-sided resections, the strategy was changed in 17.4% (34 of 196 patients). Compared to our right-sided resections with a strategy change in 13.2% (10 of 76 patients), we could only show a slight tendency. Further, we performed an ileocolic hand-sewn isoperistaltic side-to-side anastomosis in our right hemicolectomies, in the literature Morales performed a stapled one [26].

Even though a strategy change can be regarded as a reference for the impact of ICG-FA, the proof of a correlation between strategy change and avoided AL is still missing. If perfusion would be the only factor influencing anastomotic healing, after a strategy change and thus perfect perfusion, no AL should happen. Our data for right hemicolectomies and LAR with TME show a tendency that patients where a strategy change was undertaken were suffering from less AL. Even though many other studies report a positive effect of ICG-FA on the AL rate, this potentially positive effect rate has to be proven by randomized controlled multicenter studies in the future [9], [27], [28], [29], [30], which are already ongoing (ICG-COLORAL, IntAct). Despite that fact and based on our first positive results [1] ICG-FA was established as standard procedure for the assessment of bowel perfusion and determination of the resection line for colorectal resections in our clinical practice.

Already 2010 Kudszus et.al. [31] showed a lowering of the AL rate by a factor 2 from 7.5% (control group) to 3.5% in the ICG-FA group. Jafari [27] reported on 40 patients with robotically assisted LAR (16 with ICG-FA and 24 in the control group), where he observed a threefold reduced AL rate of 6% in the ICG-FA group vs. 18% in the control group. The prospective multicenter PILLAR II study analyzed 139 patients in 2014 after colorectal resections for benign or malignant disease. Even the rectal resection group was inhomogeneous, as only in 81% the left flexure was lowered and only in 61% a high tie of the AMI was performed. The authors report a very low AL rate of 1.4% for the ICG-FA group [27]. In another study [29], ICG-FA lead to proximal change of the transection line by more than 5 mm in 26.5% of patients. In those patients only three AL occurred (4.4%).

In contrast to those reported publications, we investigated our homogeneous group of patients, who were all equally treated in terms of the used operative technique and perioperative protocols. The only difference between the groups was the use of ICG-FA. Our data show a threefold lowering of the AL rate for patients with LAR, which resembles the results of Jafari et al [32]. The result was statistically not significant. This could be due to the small sample size or more likely to a selection bias by nonevaluated factors.

As our right hemicolectomies are strictly following the operative protocol with CME, which was shown to be superior in comparison with non-CME standards regarding lower local recurrence rate and higher 5-year survival rates [33], we are evaluating a homogenous collection of patients for statistical evaluation. The ileotranversostomy was always performed in a side-to-side, isoperistaltic handsewn manner. ICG-FA was leading to a slightly higher change rate of intraoperative strategy (17.1%), compared to our above evaluated rectal resections. As one of our patients developed an AL of grade C, even though the strategy was changed, the protective effect of this change for AL has to be discussed for right hemicolectomies. Finally, only one AL occurred in the ICG-FA group, leading to an AL rate of 1.3%. Compared to our control group, the AL rate was lowered by a factor of 6 compared to the non-ICG-FA group (8.1% AL rate). This result was statistically significant. We have to admit that the control group contained significantly more patients with ASA III and IV, reflecting more seriously ill patients with a higher risk for AL. Compared to the literature, this result was surprising, as we could show a higher impact of ICG-FA on our end point, the AL rate, especially in patients with right hemicolectomies.

Finally, looking at our statistical evaluation on a series of patients, compared to our own control group, a selection bias by nonevaluated factors should be taken into account. Only a prospective randomized study with equal control groups or a matched pair analysis could finally exclude a selection bias.

Until today, ICG-FA represents an additional qualitative tool to assess bowel perfusion in abdominal surgery. Like the visual assessment is strongly dependent on individual experience, the additional information from ICG-FA has to be carefully assessed by the surgeon on the screen. Thus, only taking the latest intensity of green into account might lead to false decisions, as venous obstruction could favor accumulating ICG in the bowel wall. The color coding of the ICG intensity can be helpful, as it gives additional information about ICG flooding and thus arterial perfusion. Other groups evaluate the video data retrospectively, calculating the intensity rise as a slope of the ICG intensity curve over time [4]. New methods of real-time evaluation using artificial intelligence might close that gap in the future. As those calculations are not available in real time until now, we still help ourselves by comparing the perfusion of our anastomotic site with a central small bowel loop, which is directly perfused by the superior mesenteric artery (SMA). This gives us more information about central circulation time and intensity rise. During rectal resections or left hemicolectomies, observing this and comparing it with small bowel in the same visual section to the perfusion of the descending colon after dissection of the mesocolon just before transection gives us additional information about perfusion time through the marginal artery of Drummond. After the first phase of flooding and intensity rise, in the long run, even a minor perfused descending colon lights up intensely green, and this information is finally useless for the determination of our transection line. The impact of ICG-FA, especially on the left-sided resection [26], might be due to the delicate situation of the perfusion by only one small marginal artery (Drummond) in the descending colon, which is part of the anastomosis. Even though we could not show a significant effect on LAR, in right hemicolectomies, the effect of ICG-FA was significant. This might be due to a similar perfusion situation in the anastomotic region, especially of the terminal ileum.

Finally, the definite trend and advantage for the use of ICG-FA was shown in our colorectal resections, and our former data were confirmed. Thus, we are going on using ICG-FA routinely for any abdominal bowel resection. ICG-FA is proven to be a simple and easy to use tool, which does not prolong the operation more than 10 min. Even though it is still a subjective tool, where the surgeon visually decides on the base of different green levels, the impact is clearly shown. Further, the investigation of the anastomosis in LAR by rectoscopy under ICG-FA might give additional valuable information. It remains to be seen what possibilities for visualization and evaluation of the blood flow in various organs might be delivered by new examination methods, such as hyperspectral imaging with white light without contrast medium and fusion imaging. Finally, real-time evaluation of, e.g., intensity rise (slope) in the bowels might add up more valuable information and even lead to better safety for our patients, especially when combined with artificial intelligence.
