# Supplementary material for: Intraoperative fluorescence angiography with indocyanine green: retrospective evaluation and detailed analysis of our single-center 5-year experience focused on colorectal surgery
Source: Innov Surg Sci. 2020 Sep 18;5(1-2):35–42. doi: 10.1515/iss-2020-0009 (PMC7798305; doi:10.1515/iss-2020-0009)
Supplement: Supplementary file 1 [file iss-5-20200009-s001.pdf]

## Reviewer Assessment

# Christoph Marquardt, Georgi Kalev and Thomas Schiedeck: Intraoperative fluorescence angiography with indocyanine green. Retrospective evaluation and detailed analysis of our single center five year experience focused on colorectal surgery

### Reviewers' Comments to Original Submission

**Reviewer 1: anonymous**

Date received: 29-06-2020
Reviewer recommendation: **Return to author for minor modifications**
Reviewer overall scoring: **High**

Assessment Form scores: 5 = High/Yes; 3 = Medium/Adequate; 1 = Low

| | | | | | |
|---|---|---|---|---|---|
| Is the subject area appropriate for the journal | | 4 | | | |
| Does the title clearly reflect the paper's content? | | 4 | | | |
| Does the abstract clearly reflect the paper's content | | 4 | | | |
| Do the keywords clearly reflect the paper's content? | | 4 | | | |
| Does the introduction present the problem clearly? | | 4 | | | |
| Are the results/ conclusions justified? | | 4 | | | |
| How comprehensive and up-to-date is the subject matter presented? | | 4 | | | |
| How adequate is the data presentation? | | 4 | | | |
| Are units and terminology used correctly? | | 4 | | | |
| Is the number of cases adequate? | | 4 | | | |
| Are the experimental methods/ clinical studies adequate? | | 4 | | | |
| Is the length appropriate in relation to the content? | | | | 2 | |
| Does the reader get new insights from the article? | | | 3 | | |
| Please rate the practical significance. | | 4 | | | |
| Please rate the accuracy of methods. | | 4 | | | |
| Please rate the statistical evaluation and quality control. | | 4 | | | |
| Please rate the appropriateness of the figures and tables. | | | 3 | | |
| Please rate the appropriateness of the references. | | 4 | | | |
| Please evaluate the writing style and use of language. | | | 3 | | |
| Please judge the overall scientific quality of the manuscript. | | 4 | | | |

| | |
|---|---|
| Are the methods used worthy of reproduction in greater deal? | yes |

**Comments to author:** This manuscript is addressing an actual issue: the evaluation of the blood supply in the area of a digestive anastomosis. The authors describe a large series with convincing results and a clear statement regarding the use of intraoperative fluorescence. There are some comments that might facilitate the reader to get into the details of this work:

1. The abstracts could be shortened by presenting only LAR and right colectomies. The additionally described procedures are not relevant for the abstract and the abstract is quite long.
2. The authors could state why the avoidance of using ostomies was increased by ICG-FA because at the end, the healing of the anastomosis is influenced by the blood supply and not by the presence of an ostomy (even though the consequences are different, if an AL occurs).
3. An accurate analysis of the factors that caused AL in their patients would it helpful, with emphasis on necrosis or insufficient blood supply.
4. One would have expect the LAR to be at higher danger for impaired blood supply compared to RC, do the authors have an explanation why this was somehow different in their study?
5. A table with literature data on the use of ICG, change of strategy and AL rate including own data would be helpful and keep the discussion shorter.
6. The long discussion is including redundant information that has been described in the part results, it may be helpful to delete it here.
7. Where there any additional information on positive lymph nodes during ICG (outside of the usual resection margin), meaning that the resection had to be extended (knowing that this was not the purpose of this study)
8. It would be nice, if the authors could give us an insight on possible future aspects (other technologies, the use of white light and fusion imaging etc).

**Reviewer 2: anonymous**

Date received: 7-Jul-2020
Reviewer recommendation: **Accept in present form**
Reviewer overall scoring: **Excellent**

Assessment Form scores: 5 = High/Yes; 3 = Medium/Adequate; 1 = Low

| | | | | | |
|---|---|---|---|---|---|
| Is the subject area appropriate for the journal | 5 | | | | |
| Does the title clearly reflect the paper's content? | 5 | | | | |
| Does the abstract clearly reflect the paper's content | | 4 | | | |
| Do the keywords clearly reflect the paper's content? | 5 | | | | |
| Does the introduction present the problem clearly? | 5 | | | | |
| Are the results/ conclusions justified? | | 4 | | | |
| How comprehensive and up-to-date is the subject matter presented? | | 4 | | | |
| How adequate is the data presentation? | | 4 | | | |
| Are units and terminology used correctly? | 5 | | | | |
| Is the number of cases adequate? | | | 3 | | |
| Are the experimental methods/ clinical studies adequate? | | 4 | | | |
| Is the length appropriate in relation to the content? | 5 | | | | |
| Does the reader get new insights from the article? | | 4 | | | |
| Please rate the practical significance. | | 4 | | | |

| | | | | | |
|---|---|---|---|---|---|
| Please rate the accuracy of methods. | | 4 | | | |
| Please rate the statistical evaluation and quality control. | 5 | | | | |
| Please rate the appropriateness of the figures and tables. | | 4 | | | |
| Please rate the appropriateness of the references. | 5 | | | | |
| Please evaluate the writing style and use of language. | 5 | | | | |
| Please judge the overall scientific quality of the manuscript. | | 4 | | | |
| Are the methods used worthy of reproduction in greater deal? | Yes | | | | |

**Comments to author:** This excellent article presents with clear methods and results the impact of ICG-FA on strategy change in resection due to bowel perfusion as shown in former publications. This assumed positive effect needs to be proven in RCT's as results might have an bias. Furthermore technical advancements for (semi-)quantitative measurements are necessary to avoid insecure visual estimation of perfusion quality using ICG.

## Authors' Response to Reviewer Comments

Date received: 17-07-2020

**Response to reviewer 1**

Reviewer 1: Thank you for your very helpful comments to our manuscript, which we included in our revised version point by point:

1. We shortened the abstract and erased all other additionally described procedures except LAR and right hemicolectomies.

2. Due to the literature, we stated in our publication, that a protective diverting ileostomy was regularly planned in any LAR, as it was shown to prevent AL after LAR [21, 22] and severe septic complications and operative revisions due to AL were reduced [23]. We added: *Due to our national guidelines and in our understanding, the above mentioned anastomotic leakage rates represent a high risk for the patient, which has to be lowered by any means, e.g. performing a protective ileostomy.*

3. In our retrospective analysis of the clinical charts and operation reports from patients with anastomotic leakage, we could not extract valid data in regard to necrosis or perfusion. Besides we did not use ICGFA to analyze the situation of the anastomosis before revision. We only used ICGFA after resection of the leakage and before performing the new anastomosis.

4. Special thanks for this remark! Reviewing our data deeply again, we have to admit, that our strategy changes due to impaired blood supply were according to the literature slightly lower in right colon resections. We corrected our text and appended: Summing up our left sided resections, the strategy was changed in 17,4% (34 of 196 patients). Compared to our right sided resections with a strategy change in 13,2% (10 of 76 patients), we could only show a slight tendency. Further we performed a ileo-colic hand-sewn isoperistaltic side – to – side anastomosis in our right hemicolectomies, in the literature Morales performed a stapled one [26].

5. As we added the above mentioned information to the discussion now and we are not expecting further statistically significant information, we would like to avoid another table.

6. We erased the redundant information from our discussion.

7. From our standard staging CT or MRI scans, there was no additional lymph nodes visible in our patients.

8. We agree to your comment and added the following sentence to our conclusions: It remains to be seen what possibilities for visualisation and evaluation of the blood flow in various organs might be delivered by new examination methods, such as hyperspectral imaging with white light without contrast medium and fusion imaging.

**Response to reviewer 2**

Reviewer 2: Thank you very much for your approval and comments.

## Reviewers' Comments to Revised Submission

**Reviewer 1: anonymous**

Date received: 28-07-2020
Reviewer recommendation: **Accept in present form**
Reviewer overall scoring: **High**

Assessment Form scores: 5 = High/Yes; 3 = Medium/Adequate; 1 = Low

| | | | | | |
|---|---|---|---|---|---|
| Is the subject area appropriate for the journal | | 4 | | | |
| Does the title clearly reflect the paper's content? | | 4 | | | |
| Does the abstract clearly reflect the paper's content | | 4 | | | |

| | | | | | |
|---|---|---|---|---|---|
| Do the keywords clearly reflect the paper's content? | | 4 | | | |
| Does the introduction present the problem clearly? | | 4 | | | |
| Are the results/ conclusions justified? | | 4 | | | |
| How comprehensive and up-to-date is the subject matter presented? | | 4 | | | |
| How adequate is the data presentation? | | 4 | | | |
| Are units and terminology used correctly? | | 4 | | | |
| Is the number of cases adequate? | | 4 | | | |
| Are the experimental methods/ clinical studies adequate? | | 4 | | | |
| Is the length appropriate in relation to the content? | | 4 | | | |
| Does the reader get new insights from the article? | | 4 | | | |
| Please rate the practical significance. | | 4 | | | |
| Please rate the accuracy of methods. | | 4 | | | |
| Please rate the statistical evaluation and quality control. | | 4 | | | |
| Please rate the appropriateness of the figures and tables. | | 4 | | | |
| Please rate the appropriateness of the references. | | 4 | | | |
| Please evaluate the writing style and use of language. | | 4 | | | |
| Please judge the overall scientific quality of the manuscript. | | 4 | | | |
| Are the methods used worthy of reproduction in greater deal? | yes | | | | |

**Comments to author:** Thanks to the authors for the modifications that have been undertaken and congratulations for this accurate performed study.

**Reviewer 2: anonymous**

Date received: 09-Aug-2020
Reviewer recommendation: **Accept in present form**
Reviewer overall scoring: **Excellent**

Assessment Form scores: 5 = High/Yes; 3 = Medium/Adequate; 1 = Low

| | | | | | |
|---|---|---|---|---|---|
| Is the subject area appropriate for the journal | 5 | | | | |
| Does the title clearly reflect the paper's content? | 5 | | | | |
| Does the abstract clearly reflect the paper's content | | 4 | | | |
| Do the keywords clearly reflect the paper's content? | 5 | | | | |
| Does the introduction present the problem clearly? | 5 | | | | |
| Are the results/ conclusions justified? | | 4 | | | |
| How comprehensive and up-to-date is the subject matter presented? | | 4 | | | |
| How adequate is the data presentation? | | 4 | | | |
| Are units and terminology used correctly? | 5 | | | | |
| Is the number of cases adequate? | | | 3 | | |
| Are the experimental methods/ clinical studies adequate? | | 4 | | | |
| Is the length appropriate in relation to the content? | 5 | | | | |
| Does the reader get new insights from the article? | | 4 | | | |

| | | | | | |
|---|---|---|---|---|---|
| Please rate the practical significance. | | 4 | | | |
| Please rate the accuracy of methods. | | 4 | | | |
| Please rate the statistical evaluation and quality control. | 5 | | | | |
| Please rate the appropriateness of the figures and tables. | | 4 | | | |
| Please rate the appropriateness of the references. | 5 | | | | |
| Please evaluate the writing style and use of language. | 5 | | | | |
| Please judge the overall scientific quality of the manuscript. | | 4 | | | |
| Are the methods used worthy of reproduction in greater deal? | Yes | | | | |

**Comments to author:** Accept as it is.
